# L-4, a Well-Tolerated and Orally Active Inhibitor of Hedgehog Pathway, Exhibited Potent Anti-tumor Effects Against Medulloblastoma *in vitro* and *in vivo*

**DOI:** 10.3389/fphar.2019.00089

**Published:** 2019-02-21

**Authors:** Mingfei Zhu, Hong Wang, Chenglin Wang, Yanfen Fang, Tong Zhu, Weili Zhao, Xiaochun Dong, Xiongwen Zhang

**Affiliations:** ^1^Shanghai Engineering Research Center of Molecular Therapeutics and New Drug Development, School of Chemistry and Molecular Engineering, East China Normal University, Shanghai, China; ^2^Department of Medicinal Chemistry, School of Pharmacy, Fudan University, Shanghai, China

**Keywords:** L-4, hedgehog, Smo, medulloblastoma, D473H

## Abstract

Inhibition of aberrant Hedgehog (Hh) pathway had been proved to be a promising therapeutic intervention in cancers like basal cell carcinoma (BCC), medulloblastoma (MB), and so on. Two drugs (Vismodegib, Sonidegib) were approved to treat BCC and more inhibitors are in clinical investigation. However, the adverse effects and drug resistance restricted the use of Hh inhibitors. In the present study, 61 synthesized compounds containing central backbone of phthalazine or dimethylpyridazine were screened as candidates of new Hh signaling inhibitors by performing dual luciferase reporter assay. Among the compounds, L-4 exhibited an IC_50_ value of 2.33 nM in the Shh-Light II assay. L-4 strongly inhibited the Hh pathway *in vitro* and blocked the Hh pathway by antagonizing the smoothened receptor (Smo). Remarkably, L-4 could significantly suppress the Hh pathway activity provoked by Smo mutant (D473H) which showed strong resistant properties to existing drugs such as Vismodegib. Orally administered L-4 exhibited prominent dose-dependent anti-tumor efficacy *in vivo* in Ptch+/-; p53-/- MB allograft model. Furthermore, L-4 showed good tolerance in acute toxicity test using ICR mice. These evidences indicated that L-4 was a potent, well-tolerated, orally active inhibitor of Hedgehog pathway, and might be a promising candidate in development of Hh-targeted anti-cancer drugs.

## Introduction

The Hedgehog pathway is evolutionarily conserved and plays vital roles in the early development of the mammals by controlling cell proliferation and differentiation (Pomeroy et al., 2002; Jiang and Hui, 2008). It was found in a state of suppression in most adult tissues and only involved in the maintenance and repair of some tissues (Rimkus et al., 2016). The main components of the Hh pathway in mammals include the following parts: three Hh ligands (Sonic hedgehog, Indian hedgehog, Desert hedgehog) which can stimulate the Hh pathway, PTCH which represses the activity of Smo, Smo which acts as a positive regulatory protein, and the GLI family (Ruiz and Altaba, 1997). PTCH and Smo are transmembrane proteins (Roudaut et al., 2011). In the absence of Hh ligands, PTCH inhibits Smo activity by repressing its trafficking and localization to the cilia (Cheng and Bishop, 2002; Goetz and Anderson, 2010). Once the Hh ligands bind to PTCH, PTCH would leave the cilia and Smo would be able to accumulate and activate in the cilia (Sharpe et al., 2015; Zhang et al., 2017). Activated Smo subsequently starts a downstream signaling leading to the activation of transcription factors, GLI. Then, the activated GLIs translocate into the nucleus to induce the expression of various context-specific genes which regulate cellular differentiation, proliferation and survival (Scales and de Sauvage, 2009; Stecca and Ruiz, 2010).

Aberrant activation of Hh pathway signaling leads to abnormal development of the tissues and is linked to many malignant tumors like BCC, MB, and so on (Epstein, 2008; Rimkus et al., 2016; Tan et al., 2018). MB, known as a primitive neuroectodermal tumor, is the most common malignant pediatric brain tumor (Romer et al., 2004). It happens in the cerebellum and accounts for about 20% of all pediatric brain tumors (Ellison et al., 2003). Previous studies had shown that Hh pathway was closely related to MB. Mutant PTCH1 was reported to cause the human developmental disorder Gorlin syndrome which increased a notable risk of advanced BCC and MB. This indicated that Hh pathway contributed to the formation of MB (Hahn et al., 1996). Actually, further researches showed abnormal expression of PTCH1, SMO, and SUFU in a large percentage of spontaneous MB (Epstein, 2008; Kool et al., 2008). Up to now, MB are termed four subgroups: WNT, SHH, group 3, and group 4 (Taylor et al., 2012). Among them, Hh pathway subtype accounts for 30% of MB (Kool et al., 2012; Korshunov et al., 2012). Thus, research and development of inhibitors targeting the Hh pathway might be a promising strategy for MB therapy (Rimkus et al., 2016).

Cyclopamine is the first Hh inhibitor to be found which advances our research of the Hh pathway as a therapeutic target substantially (Incardona et al., 1998; Tremblay et al., 2009). It is an allosteroid alkaloid extracted from the veratrine (Incardona et al., 1998). Cyclopamine could directly combine with the transmembrane domain of Smo proteins and prevent the conformational change and activation of Smo (Chen et al., 2016). However, the poor potency and oral solubility limit its application in clinical therapy (Tremblay et al., 2009). In recent years, great effort has been taken predominantly on targeting Smo. So far, a variety of Smo inhibitors have been reported. Among them, Vismodegib (GDC-0449) (Robarge et al., 2009; Kumar et al., 2017) and Sonidegib (NVP-LDE225) (Pan et al., 2010; Jalili et al., 2013) have been approved by FDA for the treatment of BCC. However, Vismodegib and Sonidegib showed undesirable adverse effect and drug resistance subsequently in clinic therapy (Scales and de Sauvage, 2009). According to the research, there are 15–33% untreated BCC patients harbor Smo mutations and the percentage increases to 69–77% in resistant tumors (Dong et al., 2018). The MB relapsed after a few months of Vismodegib treatment and it was found that the mutation of Smo D473H gave rise to the drug resistance (Yauch et al., 2009). Thus, the binding ability of Hh inhibitors to D473H is important to overcome the drug resistance.

NVP-LEQ506 (Peukert et al., 2013) and Taladegib (LY-2940680) (Bendell et al., 2018) are two representative Hh inhibitors in clinic trial. NVP-LEQ506 was developed by Novartis and LY-2940680 was developed by Lilly United States and Ignyta. LY-2940680 and Anta XV (lead compound of NVP-LEQ506) are both benzylphthalazine derivatives which were reported to be potent Hh pathway inhibitors with low nanomolar affinity for Smo. Based on these previous studies, we designed and synthesized a series of small molecular compounds containing central backbone of phthalazine or dimethylpyridazine as candidates of new Hh signaling inhibitors. The piperazine linker of lead compound Anta XV was replaced by piperidin-4-amine moiety and the benzyl group was optimized with different aromatic rings to generate compounds series A. We applied the similar strategy to replace the 4-methylamino-piperidine linker of LY-2940680 with pyrrolidin-3-amine moiety and the 1-methyl-1H-pyrazol group with benzene ring to generate compounds series L. Furthermore, in some compounds, the bicyclic phthalazine core was replaced with dimethylpyridazine to mimic the pharmacophore of NVP-LEQ506. After screening for inhibitive effects on Hh signaling pathway of these compounds, L-4 was found to be the most potent compound. Herein, evaluation of anti-tumor effects of L-4 *in vitro* and *in vivo* was conducted.

## Materials and Methods

### Reagents

Vismodegib, Taladegib, and SAG were purchased from Selleck (TX, United States). NVP-LEQ506 was bought from MedChemExpress (NJ, United States). L-4 was synthesized in-house. For *in vitro* experiments, compounds were dissolved in DMSO (Beyotime, Shanghai, China) and stock solutions were stored at -20°C. For *in vivo* experiments, Vismodegib, Taladegib and L-4 were formulated in 0.5% methylcellulose (Aladdin, Shanghai, China).

### Cell Lines and Cell Culture

Shh-Light II cell line was a gift of Professor. Philip Beachy in Stanford University and cells were maintained in DMEM (Hyclone, UT, United States) containing 10% fetal bovine serum (FBS) (Biological Industries, Israel), zeocin (Invitrogen, CA, United States) 0.15 mg/mL and G418 (Invitrogen) 0.4 mg/mL at 37°C in a 5% CO_2_ atmosphere. HEK293 human epithelial kidney cell was purchased from Cell Bank of China Science Academy (Shanghai, China) and cells were maintained in DMEM containing 10% FBS, streptomycin 100 μg/mL and penicillin 100 U/mL as recommended at 37°C in a 5% CO_2_ atmosphere.

### Animals

All animal (purchased from Shanghai SLAC Laboratory Animal Co., Ltd., Shanghai, China) care and experimental protocols for this study complied with the Chinese regulations and the Guidelines for the Care and Use of Laboratory Animals drawn up by the National Institutes of Health (USA) and were approved by the Institutional Animal Care and Use Committee of the East China Normal University. ICR mice and BALB/c nude mice (6–8 weeks old) were purchased from the Shanghai SLAC Laboratory Animal CO., LTD., Mice were maintained on a 12:12 light-dark cycle in a temperature-controlled (21∼23°C) and SPF conditional room. Mice were provided with standard rodent chow and water *ad libitum*. All animals were acclimatized for a week before beginning the study.

### Gli-Luc Reporter Assay

Shh-Light II cells were seeded in 96-well plants at a density of 4000 cells/200 μL. After 48 h of various compounds treatment as indicated, the luciferase activity was examined with Dual-Luciferase Assay Reporter System (Promega, Madison, WI, United States) following the manufacturer’s instructions. The firefly luciferase values were normalized to Renilla values. GraphPad Prism software was used to generate dose–response curves and IC_50_ values with five parameters logarithmic non-linear regression fitting.

### Medulloblastoma Primary Cells Culture and Brdu Assay

Ptch+/-; p53-/- mice were gained from hybridization of Ptch+/- mice and p53-/- mice. p53-/- mice were kindly provided by Professor Wei Zhang (East China Normal University, Shanghai, China) and Ptch+/- mice were bought from Model Animal Research Center of Nanjing University (Nanjing, China). The primary spontaneously MB in Ptch+/-; p53-/- mice were collected and allografted into BALB/c nude mice. After development in the nude mice, tumors were harvested. The cells were maintained in Neurobasal A medium (Invitrogen) containing B-27 supplement (Invitrogen), EGF 20 ng/ml (Invitrogen), bFGF 20 ng/ml (Invitrogen), non-essential amino acids (Invitrogen), N-acetylcysteine 60 μg/ml (Sigma-aldrich, St. Louis, MO, United States).

Medulloblastoma cells were seeded in 96-well plates at a density of 20000 cells/200 μL. After treated with various concentrations of L-4 for 36 h, the Brdu assay was conducted using the Brdu Cell Proliferation Kit (Abcam, Cambridge, United Kingdom) according to manufacturer’s instructions.

### Microscopy Analysis of Fluorescent BODIPY-Cyclopamine Competition Assay

Human wild type Smo (hSmo) expression vector is a gift from Professor Ke Yu (Fudan University, Shanghai, China). Microscopy analysis of fluorescent BODIPY-cyclopamine competition assay was performed as following. In brief, the HEK293 cells were seeded in 6-well plates and transfected with wild type hSmo expression vector. Two days after transfection, the cells were seeded in 24-well plates and incubated with compounds. After incubation for 10 h, the cells were washed with PBS twice, fixed with 4% paraformaldehyde in dark for 10 min, washed with PBS again and incubated with Triton X-100 solution for 10 min. The cells were then stained with DAPI (Sigma-aldrich) and photographed using a fluorescence microscope.

### Flow Cytometry Analysis of Fluorescent BODIPY-Cyclopamine Competition Assay

Fluorescent BODIPY-cyclopamine competition assay was adopted as following. In brief, HEK293 cells were seeded in 6-well plates and transfected with wild type hSmo expression vector. Two days later, cells were seeded in 24-well plates and treated with the compounds. After incubation for 10 h, the cells were harvest and tested by flow cytometry. The green fluorescence of BODIPY-cyclopamine bound to Smo was examined by a flow cytometer (Guava EasyCyte 6HT-2 L, Merck Millipore, Billerica, MA, United States). At least 5000 cells were analyzed for each data point. FACS data were analyzed with InCyte software.

### Antagonizing Drug-Resistant Assay

Drug-resistant D473H Smo expression vector is a gift from Professor Ke Yu (Fudan University, Shanghai, China). Shh-Light II cells were transfected with wild type and mutant D473H Smo expression vectors. Then the cells were seeded in 96-well plates and treated with Vismodegib and L-4 at various concentrations. After incubation for 48h, a dual luciferase reporter assay (Promega, Madison, WI, United States) was performed following the manufacturer’s instructions. 293T cells transfected with mutant Smo D473H were treated with Vismodegib and L-4 for 48 h, then cells were harvested and subjected to WB analysis.

### Molecular Modeling

Molecular docking was carried out by using Glide module in Schrödinger 2013 software package. The crystallographic structure of the human Smo 7TM receptor was retrieved from the RCSB protein data bank (ID: 4JKV). The SWISS-MODEL online server was employed to construct the mutant D473H by replace the specific residue Asp473. The WT and D473H structures were minimized by using AMBER16 software and then were prepared by using the Protein Preparation Wizard module. Two ligands were selected in the docking study: L4 and Vismodegib. Before docking, they have been processed by using the Ligprep module in Schrödinger 2013. These two molecules were docked into the binding pocket of the WT and D473H protein structure. The best docked poses were select for further analysis.

### Western Blot Analysis

The proteins of whole cell lysates were prepared in RIPA buffer containing protease and phosphatase inhibitors (Thermo Fisher Scientific, Waltham, MA, United States), and quantified by the BCA method. Immunoblotting analysis of Gli1 and Ptch1 were performed as previously described (Dorward et al., 2016). The expression of β-actin was used as loading control. Antibodies against Smo and β-actin were purchased from Santa Cruz Biotechnology (CA, United States). Antibody against Ptch1 was purchased from Abcam. Antibody against Histone H3 was purchased from CST (Danvers, MA, United States).

Cell fractionation was performed as following. The collected cells were lysed by cytoplasmic lysis buffer [50 mM Tris-HCl (PH 7.4), 140 mM NaCl, 1.5 mM MgCl_2_, and 0.5% NP-40 (1.06 g/ml)] for 20 min. Then the lysate was centrifuged for 10 min at 3000 rpm at 4°C and the supernatant was collected as cytoplasmic lysate. The pellet was washed once by cytoplasmic lysis buffer and dissolved by RIPR buffer (Thermo Fisher Scientific, Waltham, MA, United States) for 20 min. Then the lysate was centrifuged for 10 min at 12000 rpm at 4°C and the supernatant was collected as nuclear lysate.

### Medulloblastoma Allograft Model

The primary intracranial MB spontaneously developed in Ptch+/-; p53-/- mice were harvested and subcutaneously allografted into athymic nude mice. The tumors in nude mice were further collected and cut into 1 mm^3^ fragments. Then the fragments were inoculated subcutaneously into the right flank of other nude mice. When the tumor volume reached 150–200 mm^3^, the mice were randomized grouped into control and treatment groups to receive treatment accordingly. L-4 was orally administered at 5, 10, and 20 mg/kg once a day for 13 days. Taladegib was used as a positive control and orally administered at 20 mg/kg once a day. The tumor growth was recorded with the measurement of length (L) and width (W) by caliper every other day and calculated as tumor volume (V) = L × W^2^/2. Meanwhile, the body weights of mice were recorded. The TGI was calculated as TGI% = [1-(mean tumor volume of the treated group on the last day-mean tumor volume of the treated group on day 0)/(mean tumor volume of the control group on the last day-mean tumor volume of the control group on day 0)] × 100%. The tumor regression of individual mouse was defined as the tumor volume at the end was less than the tumor volume when treatment was initiated.

### Acute Toxicity Test

According to guideline 425 Organisation for Economic Co-operation Development [OECD], 2008, L-4 was administered in a single dose of 2000 mg/kg by oral in five female ICR mice (*n* = 5). A negative control was established by administering a 0.5% CMCNa solution to a female mouse (*n* = 1). Body weight of the animals were recorded shortly before the administration of the L-4 and then daily, during the treatment period. After administration, animals were observed individually during the first 30 min and then daily for 14 days. Observations included mortality and changes in skin and fur, eyes and mucous membranes, and also respiratory, circulatory, autonomic and central nervous systems, and somatomotor activity and behavior pattern. Attention should be directed to observations of tremors, convulsions, salivation, diarrhea, lethargy, sleep, and coma.

### Statistical Analysis

The statistical significance of differences between groups was evaluated by the unpaired Student’s *t* test and indicated with ^∗∗^*P* < 0.01, ^∗^*P* < 0.05. All statistical tests were two sided.

## Results

### Screening Hh Signaling Inhibitory Activities of the Synthesized Compounds

**FIGURE 1:**
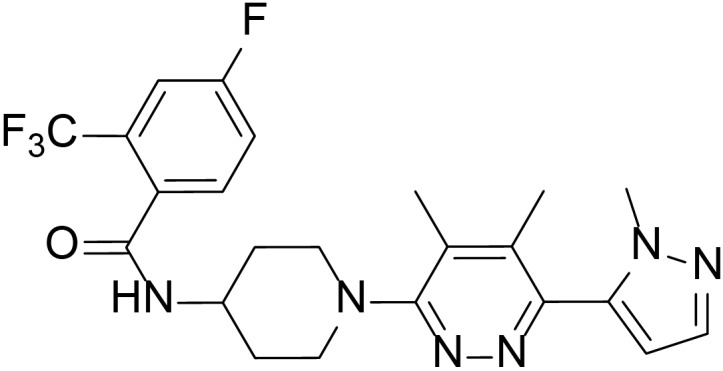
Structure of L-4.

To evaluate the inhibitory effects of all the compounds, dual luciferase reporter assays were conducted using Shh-Light II cells, which were NIH-3T3 cells stably fused with a Gli-responsive firefly luciferase and Renilla-luciferase (Xin et al., 2014). As shown in Table 1, 61 compounds were screened and 14 compounds showed good inhibitory potency. Then, we selected 5 representative compounds to further check the *in vitro* concentration-inhibition IC_50_ values. NVP-LEQ506 and LY-2940680 were used as positive controls. The IC_50_ values ranged from 1 to 50 nM (Table 2). L-4 (Figure 1) showed the best inhibitory potency with an IC_50_ of 2.33 nM.

**Table 1:** Inhibitory rate of compounds to the Hh pathway tested by dual luciferase reporter assays in Shh-Light II cells.

Compound (30 nM)	First screening inhibitory rate (%)	Second screening inhibitory rate (%)	Compound (30 nM)	First screening inhibitory rate (%)	Second screening inhibitory rate (%)
A-1	-7.04	20.15	L-7	61.61	61.17
A-2	30.32	34.48	L-8	46.78	27.97
A-3	25.85	47.63	L-9	106.47	95.84
A-4	18.11	17.66	L-10	81.75	63.77
A-5	30.52	37.46	L-11	77.72	88.68
A-6	9.27	-23.23	L-12	45.68	52.01
A-7	32.16	17.73	L-13	60.13	83.02
A-8	64.04	60.00	L-14	42.59	50.88
A-9	52.99	35.33	L-15	50.17	67.34
A-10	52.09	40.40	L-16	54.76	71.98
A-11	57.83	50.82	L-17	34.08	64.95
A-12	68.36	41.20	L-18	-15.90	37.28
A-13	21.12	29.80	L-19	13.64	52.67
A-14	17.34	47.50	L-20	14.84	36.03
A-15	-14.65	36.85	L-21	-5.37	39.89
A-16	58.48	54.85	L-22	69.50	59.59
A-17	-13.85	0.34	L-23	71.19	68.26
A-18	31.45	27.03	L-24	-9.40	-2.67
A-19	11.29	19.64	L-25	45.58	55.74
A-20	62.98	67.09	L-26	14.60	30.87
A-21	33.79	62.53	L-27	42.37	52.25
A-22	43.88	32.58	L-28	-12.71	26.22
A-23	-	73.58	L-29	-9.52	44.36
A-24	-	46.92	L-30	13.40	-1.34
A-25	-	21.82	L-31	13.93	3.76
A-26	-	20.51	L-32	15.10	38.84
L-1	-18.49	-16.43	L-33	35.28	71.25
L-2	20.99	33.38	L-34	66.91	81.92
L-3	26.88	-17.38	L-35	-	43.60
L-4	109.69	104.42	LY-2940680	-	94.99
L-5	15.07	43.32	GDC-0449	108.02	95.33
L-6	27.29	32.60	NVP-LEQ506	96.24	-


**Table 2:** IC_50_ value of compounds to the Hh pathway tested by dual-luciferase reporter assays in Shh-Light II cells.

Compounds	Hedgehog inhibitory IC_50_ (nM, mean ± SEM)
LY-2940680	2.26 ± 0.23
NVP-LEQ506	1.23 ± 0.13
A-8	21.22 ± 3.78
L-7	48.66 ± 2.97
A-11	38.38 ± 4.10
L-4	2.33 ± 0.18
A-20	39.83 ± 4.35


### L-4 Inhibits the Proliferation of Medulloblastoma Cells With Activation of Hh Pathway Signaling

**FIGURE 2:**
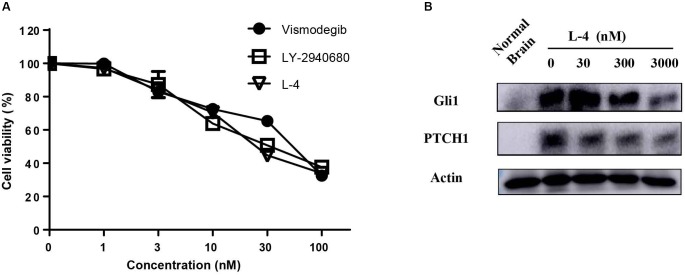
L-4 suppressed the proliferation and Hh pathway of Ptch+/-; p53-/- medulloblastoma (MB) cells *in vitro*. **(A)** L-4 inhibited the proliferation of Ptch+/-; p53-/- MB cells assessed by Brdu analysis. MB cells were exposed to various concentrations of L-4 for 36 h. Vismodegib and LY-2940680 were used as positive controls. **(B)** L-4 inhibited the expression of Gli1 and Ptch1 in primary Ptch+/-; p53-/- MB cells. MB cells were treated with various concentration of L-4 after 48 h and collected for lysis. Protein extracts were probed with specific antibodies against Gli1, Ptch1, and Actin (as a loading control). Cells isolated from normal mouse brain was marked as “Normal Brain.”

The potency of L-4 in inhibiting the proliferation of cancer cells related to activation of Hh pathway was tested. The Ptch+/-; p53-/- MB mouse model, a generally accepted Hh-driven mouse MB model which had been used to evaluate the anti-cancer effect of Smo inhibitors (Wetmore et al., 2001), was established. Spontaneous MB cells were then isolated from nude mice for testing the antiproliferative effect of compound L-4 using Brdu assay. Vismodegib and LY2940680 were used as positive controls. The results showed that L-4 dose-dependently inhibited the proliferation of MB cells in a similarity to that of Vismodegib and LY2940680 (Figure 2A). The IC_50_ of L-4 was 31.36 nM and the IC_50_ of Vismodegib and LY2940680 were 37.65 and 35.5 nM, respectively. Also, Western bolt results showed that L-4 dose-dependently inhibited the expression of Gli1 and Ptch1 in MB cells (Figure 2B).

### L-4 Binds With Smo and Restrains the Expression of Gli1 and Ptch1

**FIGURE 3:**
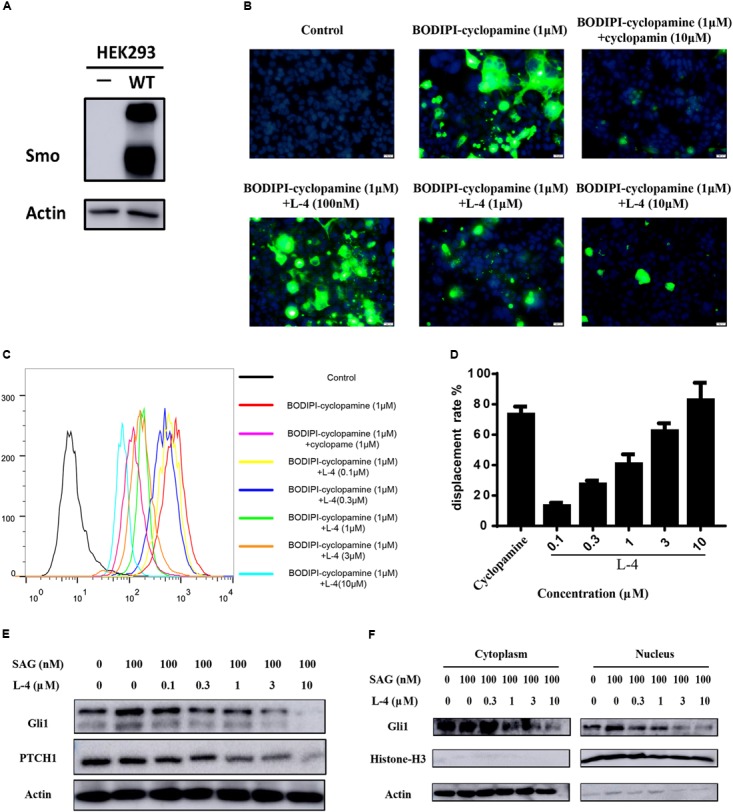
L-4 inhibited the Hh pathway by binding to Smo. **(A)** Western blot analysis of Smo expression in HEK293 cells. HEK293 cells were transfected with wild type hSmo expression vector and the Smo protein expression level was examined by western blot. Actin was used as a loading control. **(B)** BODIPI-cyclopamine competition analysis was examined by fluorescent microscope. Photographs were representatives from three independent experiments. **(C,D)** BODIPI-cyclopamine competition analysis was examined by flow cytometry. The results were expressed as mean ± SEM of three independent experiments (*n* = 3). **(E)** L-4 inhibited the expression of Gli1 and Ptch1 in Shh-Light II cells. Shh-Light II cells were treated with 100 nM SAG and various concentrations of L-4 for 48 h were harvested and subjected to WB analysis. **(F)** L-4 decreased the Gli1 expression both in the cytoplasm and the nucleus in Shh-Light II cells. The cells were treated with 100 nM SAG and various concentrations of L-4 for 48 h. After treatment, cell fractionation was performed and lysates were subjected to WB analysis.

To check whether the inhibitory potency of L-4 against the Hh pathway is due to targeting Smo, direct interaction of L-4 with Smo was analyzed by using BODIPY-cyclopamine, a kind of cyclopamine derivative with fluorescent label (Solinas et al., 2012; Sharpe et al., 2015). Firstly, HEK293 cells were transfected with the wild type Smo expression vector and the effect of transfection was examined by western blot (Figure 3A). Then, fluorescent BODIPY-cyclopamine competition assay was performed. In the presence of 10 μM L-4, cellular fluorescence of BODIPY-cyclopamine was not observed by the fluorescence microscopy analysis (Figure 3B). It was similar to the positive control, cyclopamine (Schaefer et al., 2013). As shown in Figure 3C,D, results of quantitative analysis using flow cytometry suggested that there was a dose-dependent replacement of BODIPY-cyclopamine by L-4. Taken together, our data demonstrated that L-4 was able to displace the BODIPY-cyclopamine from Smo and inhibited the Hh signaling pathway by targeting Smo. Moreover, results of western blot (Figure 3E) suggested that L-4 dose-dependently reduced the expression of Gli1 and Ptch1 in Shh-Light II cells.

It had been shown that Gli1 translocated from the cytoplasm to the nucleus to induce a series of downstream genes expression (Sharpe et al., 2015). Thus, the shuttle of Gli1 in intercellular compartments after treated with L-4 was observed. As showed in Figure 3F, SAG induced the shuttle of Gli1 to the nucleus in Shh-Light II cells. While, after treatment with different concentrations of L-4 for 48 h, a strong decrease of Gli1 both in the cytoplasm and the nucleus could be observed. The results indicated that the inhibition of Smo by L-4 not only had a negative effect on translocation of Gli1, but also inhibited the Gli1 expression.

### L-4 Inhibits Drug-Resistant Mutant Smo

**FIGURE 4:**
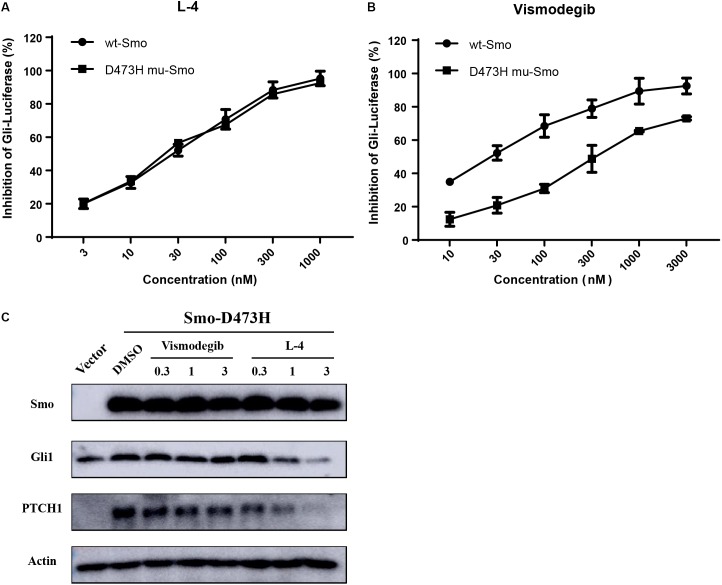
L-4 inhibited the Gli-luciferase activity stimulated by wild type Smo and mutant Smo D473H. Light II cells transfected with wild type Smo or Smo D473H plasmids were exposed to L-4 for 48 h and then were harvested for dual luciferase reporter assays. The results were expressed as the mean ± SEM. **(A)** L-4 dose-dependently inhibited the Gli luciferase activity proved by wild type Smo and mutant Smo D473H. **(B)** Vismodegib failed to inhibited the Gli luciferase activity initiated by mutant Smo D473H. **(C)** L-4 dose-dependently inhibited the expression of Gli1 and Ptch1 in 293T cells transfected with mutant Smo D473H. Transfected cells were treated with Vismodegib and L-4 for 48 h. Cells were harvested and subjected to WB analysis.

Whether L-4 could inhibit the aberrant Hh pathway provoked by mutant Smo D473H was checked. Mutant Smo D473H is an important reason for giving rise to the resistance to the current existing drugs (Dijkgraaf et al., 2011; Infante et al., 2016). Compared to the conspicuous inhibitory effect on the Shh-Light II cells stimulated by Smo wild type expression vectors, L-4 showed similar inhibitory effects on the Shh-light II cells with overexpression of mutant Smo D473H (Figure 4A). The IC_50_ values of L-4 in inhibiting wild type and D473H were 23.46 and 24.55 nM, respectively. On the contrary, the inhibiting effects of Vismodegib on Hh pathway provoked by overexpression of D473H were reduced (Figure 4B). In the present study, the IC_50_ values of Vismodegib in inhibiting wild type and D473H were 21.63 and 326.8 nM, respectively. To further confirm these results, we transfected mutant Smo D473H into 293T cells and there was a corresponding activation in Gli1 and PTCH1. Although these proteins were largely resistant to Vismodegib, they were dose dependently inhibited by L-4 (Figure 4C). To sum up, we indicate that L-4 could effectively target the mutant Smo D473H.

### The Binding Mode of L-4 With Wild Type and Mutant Smo

**FIGURE 5:**
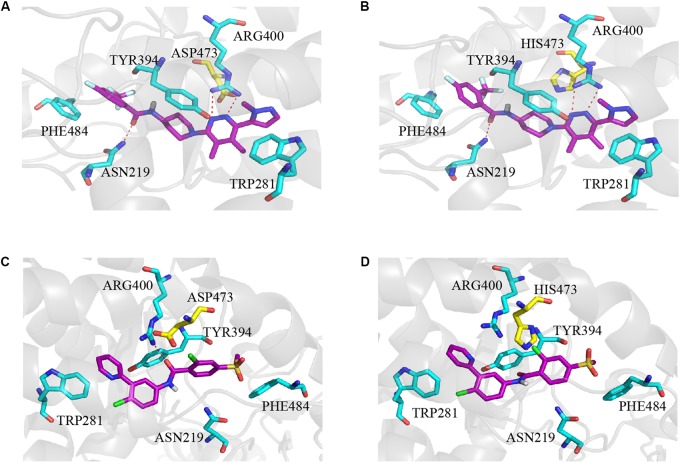
Computational predicted binding mode of L-4 and Vismodegib with wild type and mutated D473H Smo. **(A)** L-4-Smo (WT), **(B)** L-4-SMO (D473H), **(C)** Vismodegib -Smo (WT), and **(D)** Vismodegib-Smo (D473H). All pictures were generated by using Pymol.

The molecular docking simulation was used to study the binding modes of L-4 and Vismodegib with wild type and mutated D473H Smo. The predicted binding mode showed that the interaction between L-4 and wt-Smo was much stronger. As shown in Figure 5A, the inhibitor L-4 forms three critical hydrogen bonds with Smo protein: one hydrogen bond from the amide oxygen atom to Asn219 residue, two hydrogen bonds between the dimethylpyridazine core and Arg400. Furthermore, the phenyl ring and pyridazine ring also interacted with the benzene ring of Phe484 and Tyr394 via π-π stacking. It suggested the interaction between L4 and wt-Smo was much stronger. These interactions have hardly changed after the D473H mutation (Figure 5B), the distance of Hydrogen bond is changed from 2.9, 3.4, and 3.3 Å to 3.0, 3.1, and 3.4 Å, respectively. The amide oxygen atom of Vismodegib also formed one hydrogen bond with Arg400 (2.9 Å) in wt-Smo, which disappeared after D473H mutation (5.2 Å). In fact, the D473H mutation (relatively larger side chain) could change the structure of the binding pocket, especially the position of Arg400. There is only one hydrogen bond between Vismodegib and the wt-Smo, which could be easily disrupted by D473H mutation and leaded to drug resistance (Figure 5C,D). The Computational predicted binding modes were in good agreement with the experiment results.

### L-4 Inhibits the Hh Signaling Pathway Activity *in vivo*

**FIGURE 6:**
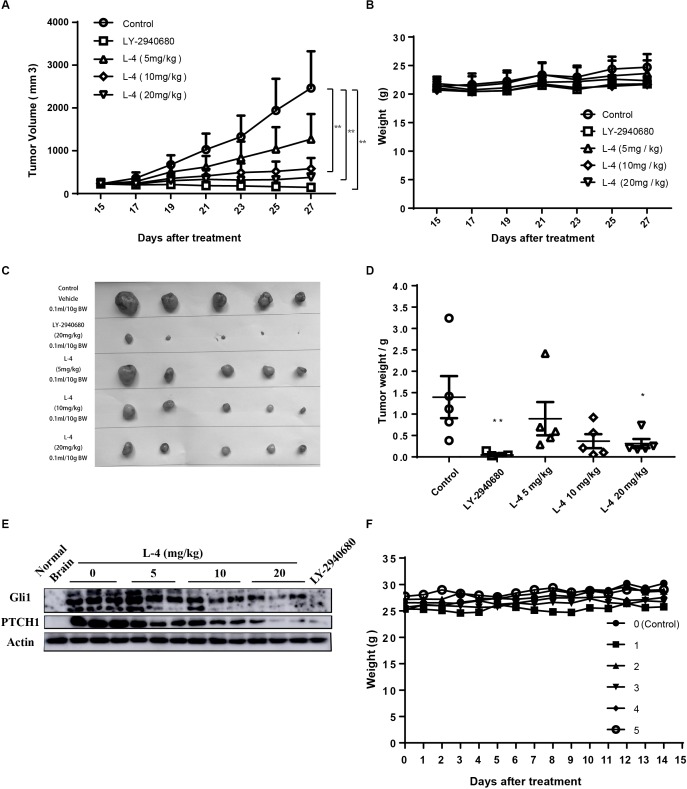
Anti-tumor activity of L-4 in Ptch+/-; p53-/- MB allograft model. Mice bearing in MB were orally administered with LY-2940680 and L-4 once a day for 13 days. **(A)** Tumor volume for indicated days was showed as mean ± SEM (*n* = 5). **(B)** Mean body weights were presented as mean ± SEM (*n* = 5). **(C)** The pictures of tumors. **(D)** Tumor weight. **(E)** L-4 inhibited the Gli1 and PTCH1 expression in the tumors. After 4 h of last dosage, tumor samples were collected for examining the expression of Gli1 and PTCH1 protein expression. **(F)** Body weight for the ICR mice in the acute toxicity test. Number 0 was the negative control mouse orally administered with 0.5% CMCNa solution. Number 1–5 were the mice orally administered with a single dose of 2000 mg/kg L-4, separately. ^∗^*p* < 0.05, ^∗∗^*p* < 0.01 vs. control.

Subcutaneous Ptch+/-; p53-/- MB allograft model was used to assess the *in vivo* anti-tumor efficacy of L-4. L-4 was orally administered at a dose of 5, 10, or 20 mg/kg once a day. As showed in Figure 6A,C,D, L-4 showed dose-dependent anti-tumor activity after 13 days of oral administration. Even at the lowest dose level of 5 mg/kg, tumor growth was significantly inhibited by L-4 compared to the vehicle control. TGI were 53.3, 84.3, and 93.3% under the dose of 5, 10, and 20 mg/kg, respectively. Body weights did not change significantly in mice treated with L4 at all doses (Figure 6B). Four hours after the last dose of vehicle, L-4 and LY-2940680 treatment, tumors were harvested and Gli1 and Pthc1 expression levels were checked as pharmacodynamic markers of Hh pathway. The results showed that L-4 could dose-dependently decrease the expression of Gli1 and Ptch1 in tumors (Figure 6E). Herein, these studies suggested that L-4 successfully induced TGI *in vivo* as it worked *in vitro* by inhibiting Hh pathway.

### L-4 Shows No Toxicity in 14-Day Test of ICR Mice

L-4 was run through acute toxicity test as part of the preclinical safety assay. In 14-day test using ICR mice, mice receiving a single dose of 2000 mg/kg L-4 did not exhibit any toxic symptom (Figure 6F). Briefly, it could be predicted that the LD_50_ of the L-4 might be higher than 2000 mg/kg.

## Discussion

The abnormal expression of Hh pathway, which has a key role in the development and maintenance of many organs and tissues, has been linked to tumorigenesis. It had been proved that aberrant in this pathway can lead to BCC and MB. Recent researches demonstrated that Hh pathway could also be linked to many other cancers, including pancreatic (Rajurkar et al., 2012; Miyazaki et al., 2016; Kumar et al., 2017), colon (Park et al., 2011), gastric (Tang et al., 2018), lung (Zhu et al., 2017), breast (Song et al., 2016), and prostate cancers (Chen et al., 2011), leukemias and multiple myeloma (Blotta et al., 2012). As a result, development of novel Hh inhibitors is of great meaning and urgency. Here, we designed and synthesized a series of small molecular compounds containing central backbone of phthalazine or dimethylpyridazine as candidates of new Hh signaling inhibitors. After evaluation with dual luciferase reporter assays using Shh-Light II cells, among the all compounds, L-4 showed the best inhibitory potency with an IC_50_ of 2.33 nM.

*In vitro*, evaluating by Brdu assay, L-4 showed obvious inhibitory effect on the proliferation of primary MB cells with nanomolar IC_50_ just as Vismodegib and Taladegib. In addition, western blot analysis of primary MB cells treated with L-4 revealed that the expression of Hh pathway were repressed. These results confirmed that L-4 had inhibition effects on proliferation of MB cells by targeting Hh pathway.

BODIPI-cyclopamine is the derivative of cyclopamine with a fluorescent tag. Those inhibitors which share the same binding domain with cyclopamine can competitively prevent BODIPI-cyclopamine from binding to Smo protein. Correspondingly, the reduce of fluorescence signal of BODIPI-cyclopamine would be detected in the microscopy and flow cytometry analysis. In this occasion, we are able to confirm the binding domain between the inhibitor and Smo. To check whether L-4 could directly bind to Smo and to find the domain of binding between L-4 and Smo, BODIPI-cyclopamine competition assay was conducted in the present study. Both the results of fluorescence microscope analysis and the results of flow cytometry analysis showed the concentration-dependent competitive relationship between L-4 and BODIPI-cyclopamine. The results suggested that L-4 could directly bind to the transmembrane domain of Smo and occupied the same pocket as cyclopamine. As previous reported, in activating Hh pathway, Gli1 shuttles from the cell cytoplasm to nucleus to start the downstream signal in nucleus (Scales and de Sauvage, 2009). Under the treatment of L-4, there was an obvious decrease of Gli1 in both cytoplasm and nucleus. The reduce of Gli1 in the nucleus might be resulted from block of the Hh pathway by L-4. Under blocking of the Hh pathway, more Gli1 protein was restrained by the SuFu, and thus few Gli1 could go through the nucleus. Since Gli1 is also a Hh target gene and significantly influenced by activation of Hh pathway, there might be a negative feedback after L-4 binds to the Smo. Less Gli1 shuttles into nucleus might lead to reduction in the gene expression of Gli1 which resulted in the decrease of expression of Gli1 protein. Therefore, the level of Gli1 in cytoplasm also decreased.

We further checked the ability of L-4 in binding with mutant Smo D473H. In the antagonizing drug-resistant assay, L-4 exhibited equivalent potency in inhibiting the Gli-luciferase activity, and downstream biomarkers provoked by Smo wild type and Smo D473H. On the contrary, Vismodegib showed ameliorated ability in inhibiting the Gli-luciferase activity provoked by Smo D473H. In 293T cells, L-4 blocked the downstream Hh pathway biomarkers driven by mutant Smo D473H while Vismodegib failed. The results suggested possible better therapeutic effects of L-4 than Vismodegib in clinic because many patients suffered from drug resistance after treated by Vismodegib. Take molecular modeling into consideration, strong hydrogen bonds between L-4 and Smo protein suggested that the binding between L-4 and Smo might be stable. Particularly, results of calculational chemistry also showed that L-4 could equally bind to the wild type and mutant Smo, which supported results of our drug-resistant study. Vismodegib showed much weaker binding ability to mutant Smo. The docking scores of Vismodegib to wild type Smo and mutant Smo were -8.62 and -7.39, respectively. The inhibition ability of Vismodegib in antagonizing mutant D473H is more than ten folds weaker than that of wild type. The results were consistent with the IC_50_ values of Vismodegib in inhibiting wild type and mutant D473H transfected in the Shh-Light II cells, which were 21.6 and 326.8 nM, respectively. The mutation (relatively larger side chain) of Smo can change the structure of the binding pocket, especially the position of Arg400. There is only one hydrogen bond between Vismodegib and the Smo which can be easily disrupted by the mutation, while the interaction between L-4 and Smo is much stronger, thus their binding is not greatly affected by the mutation.

Of note, our tumor profiling data indicated that orally administration of L-4 showed remarkable dose-dependent anti-cancer effects in the Ptch+/-; p53-/- MB allograft model without inducing loss of body weight. At the dose of 20 mg/kg, tumors in all five mice almost stopped growing in the 13-day treatment. The expression levels of Gli1 and Ptch1 in tumors reduced along with augment of the dose of L-4. It suggested that L-4 inhibited MB tumor growth by inhibiting Hh pathway and Gli1 and Ptch1 might be important pharmacodynamic markers. Besides, according to the results of acute toxicity test, L-4 was well tolerated in adult mice and no obvious side effects were observed.

**FIGURE 7:**
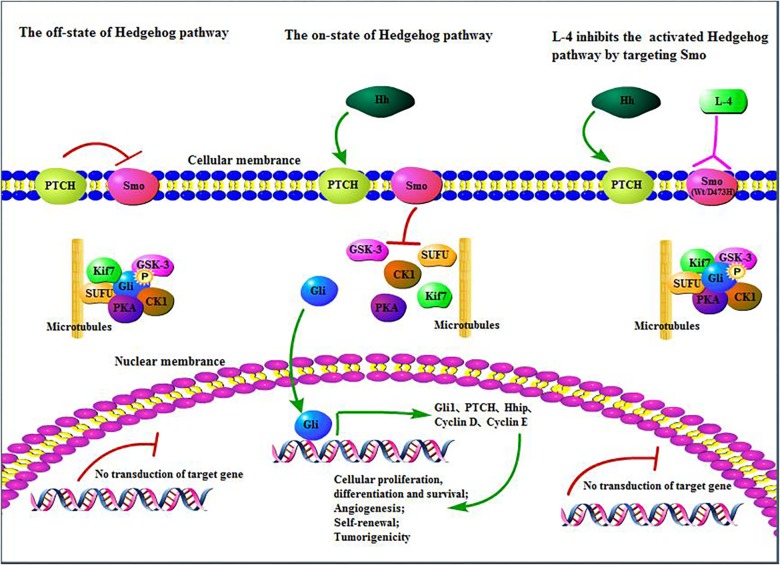
L-4 inhibited the Hedgehog pathway by targeting Smo. L-4 bound with wild type and mutant D473H Smo to prevent Gli from being released from SUFU protein complex and then inhibited the translocation of Gli into the nucleus to induce the expression of various context-specific genes which regulate cellular differentiation, proliferation, and survival.

In all, L-4 exhibited excellent drug-like properties. L-4 could bind to both wild type and mutant D473H Smo and then inhibited the escapement of Gli from SUFU protein complex and translocation of Gli into the nucleus which would induce the expression of various context-specific genes related to regulation of cellular differentiation, proliferation, and survival (Figure 7). It had considerable anti-tumor efficacy *in vitro* and *in vivo*. It was highly soluble and with limited toxicity. Nowadays, many preclinical and clinical studies are conducted to develop novel Hh inhibitors or combine Hh inhibitors with other clinical medicine in hope to cure cancers with overexpressed Hh pathway (Riobo et al., 2006; Wang et al., 2012; Amakye et al., 2013). Although previous studied Hh inhibitors failed in clinical trials in all other tumors except BCC and MB, more effort should be taken to research and develop new Hh inhibitors. Since L-4 could bind with wild type and mutant Smo in the aberrant Hh pathway, it is reasonable that L-4 could be a promising candidate as new anti-cancer agent and create more opportunities in cancer therapy.

## Author Contributions

XZ, YF, WZ, and XD conceived and designed the study. MZ, HW, and CW performed the experiments. TZ performed the molecular docking analysis. MZ wrote the manuscript. XZ, WZ, and XD supervised the whole experimental work and revised the manuscript. All authors read and approved the final manuscript.

## Conflict of Interest Statement

The authors declare that the research was conducted in the absence of any commercial or financial relationships that could be construed as a potential conflict of interest.

## Abbreviations

BCA: bicinchoninic acid
BCC: basal cell carcinoma
CMCNa: carboxymethyl cellulose sodium
GLI: glioma associated oncogene
Hh: hedgehog
ICR mice: institute of cancer research mice
MB: medulloblastoma
PTCH: patched
SHH: sonic hedgehog
Smo: smoothened
SPF: specific pathogen-free
TGI: tumor growth inhibition
WNT: wingless
